# Protective Effect of Monoterpene Isoespintanol in a Rat Model of Prediabetes Induced by Fructose

**DOI:** 10.3390/ph17010047

**Published:** 2023-12-28

**Authors:** Luciana Di Sarli Gutiérrez, María Cecilia Castro, Sherley Farromeque Vásquez, Hernán Gonzalo Villagarcía, Luisa González Arbeláez, Benjamín Rojano, Guillermo Schinella, Bárbara Maiztegui, Flavio Francini

**Affiliations:** 1 CENEXA (Centre for Experimental and Applied Endocrinology, UNLP CONICET CCT La Plata, CEAS CICPBA), School of Medicine, Street 60 and 120, La Plata 1900, Argentina; direccion-cenexa@laplata-conicet.gov.ar (L.D.S.G.); mccastro@cenexa.org (M.C.C.); sfarromeque@med.unlp.edu.ar (S.F.V.); hvillagarcia@med.unlp.edu.ar (H.G.V.); bmaiztegui@cenexa.org (B.M.); 2 CIC (Centre for Cardiovascular Research, UNLP CONICET CCT La Plata), School of Medicine, Street 60 and 120, La Plata 1900, Argentina; luisafarbelaez@med.unlp.edu.ar; 3 Food Science Laboratory, Faculty of Sciences, National University of Colombia, Medellín Campus, Medellin 050034, Colombia; brojano@unal.edu.co; 4 School of Medicine, UNLP, Street 60 and 120, La Plata 1900, Argentina; schinell@med.unlp.edu.ar; 5 Institute of Health Sciences, UNAJ-CICPBA, Av. Calchaquí 6200, Florencio Varela 1888, Argentina

**Keywords:** isoespintanol, hepatic lipogenesis, inflammation, oxidative stress, prediabetes

## Abstract

A high-fructose diet (HFD) induces murine alterations like those recorded in human prediabetes. Protective effects of isoespintanol (monoterpene isolated from Oxandra cf. xylopioides) on changes induced by HFD were evaluated. Animals were maintained for 21 days with a standard diet (C), 10% fructose (F), and F plus isoespintanol (FI, 10 mg/kg, i.p.). Glycemia, triglyceridemia, total and HDL-cholesterol, and insulin resistance index (IRX) were determined. Intraperitoneal glucose tolerance test (IGTT) was performed. In the liver, we measured glycogen, lipogenic gene expression (*SREBP-1c*, *GPAT*, *FAS*, and *CPT1*), oxidative stress (GSH and 3′-nitrotyrosine content), inflammation markers (*iNOS*, *TNF-α*, and *PAI-1* gene expression; iNOS and COX-2 protein levels), p-eNOS, p-Akt, and p-GSK3β protein levels. Isoespintanol corrected enhanced triglycerides, lipogenic genes, and IRX, and reduced HDL-cholesterol induced by HFD. Increased liver glycogen and inflammatory markers and decreased GSH, p-Akt, and p-GSK3β measured in F rats were reversed by isoespintanol, and p-eNOS/e-NOS and iNOS/GADPH ratios were normalized. Isoespintanol restored glucose tolerance (IGTT) compared to F rats. These results demonstrate for the first time that isoespintanol prevents endocrine–metabolic alterations induced by HFD in prediabetic rats. These effects could be mediated by Akt/eNOS and Akt/GSK3β pathways, suggesting its possible use as a therapeutic tool for the prevention of diabetes at early stages of its development (prediabetes).

## 1. Introduction

Type 2 diabetes (T2D) is a severe chronic metabolic disease in continuous growth, characterized by elevated blood glucose levels. It is one of the leading causes of blindness, kidney failure, heart attacks, strokes, and lower limb amputations associated with premature mortality. It is estimated that by 2045, 629 million people around the world will suffer diabetes [1]. Before T2D development, there is a state called prediabetes, characterized by insulin resistance together with impaired fasting glucose, impaired glucose tolerance, or both [2]. Progression from prediabetes to T2D can be delayed by implementing lifestyle changes (healthy diet and regular physical activity) or through pharmacological intervention [2]. It has been shown that fructose consumption has increased in the last 50 years due to the incorporation into the diet of high-fructose corn syrup (HFCS), present in industrialized products, mainly in sweetened drinks containing high amounts of fructose that are rapidly absorbed [3]. HFCS is widely used due to its low cost and high sweetening power. Therefore, high consumption of free fructose (present in HFCS) is considered a major contributor to the current epidemic of diabetes, obesity, and other metabolic disorders [4,5,6,7]. In our laboratory, we have previously demonstrated that administration of a fructose-rich diet (10% *w*/*v* in the drinking water) to normal rats for three weeks, induced several endocrine–metabolic changes, including dyslipidemia, altered glucose tolerance, insulin resistance, liver steatosis, and endocrine pancreas dysfunction [8,9,10,11,12]. These abnormalities are like those described in the metabolic syndrome in humans and, in consequence, fructose-fed rats model constitute a good and validated model for studying possible pharmacological interventions at early stages of diabetes development.

Although fruits contain fructose, they have a lower fructose content compared to an industrialized drink and, in addition, contain flavonoids, epicatechin, vitamin C, and other natural products with recognized antioxidant activity that could prevent adverse effects of fructose. Even when synthetic drugs are effective in treating diabetes, it has been shown that they may have many side effects. In this sense, medicinal plants have been used for years to treat diseases and even today there are many natural products and/or synthetic variations of their structure that are used to discover and develop novel drugs [13]. Medicinal plants have been shown to have preventive effects on chronic diseases like cancer, non-alcoholic fatty liver disease, cardiovascular disease, T2D, and metabolic syndrome. However, the effects of these compounds were not systematically investigated at the early stages of T2D development (prediabetes). Experimental results confirm that some herbal bioactive compounds (resveratrol, curcumin, berberine, anthocyanin, vegetable oils, and soluble fibers) have benefit in their efficacy for decreasing insulin-resistance, fasting blood glycemia, or fasting insulin [14]. A 12-month randomized controlled trial designed to investigate metabolic effects of the natural products in people with pre-diabetes and overweight or obesity, showed that alpha-cyclodextrin, a fiber derived from corn starch, binds triglycerides in the intestines to prevent its absorption, aiding lipid control. Additionally, a hydrolyzed ginseng extract, containing high amounts of compound K, a metabolite of ginsenosides, could ameliorate hyperglycaemia [15].

However, data from different clinical trials on the effects of ginseng on prediabetes are still inconsistent. Meta-analyses revealed that ginseng supplementation significantly reduced serum concentration of IL-6 and HOMA-IR but also increased TNF-α levels [16]. Finally, a 6-month double-blind, placebo-controlled, randomized clinical trial, designed to assess the efficacy of α-cyclodextrin and of hydrolyzed ginseng for cholesterol and glycemic control in people with prediabetes and overweight or obesity, demonstrated that both compounds were ineffective [17].

In the present work, the compound isoespintanol (2-Isopropyl-3,6-dimethoxy-5-methylphenol), a monoterpene isolated from the ethereal extract of the leaves of Oxandra cf. xylopioides, was studied. Using this compound, we previously demonstrated in different experimental models its different pharmacological activities [18,19,20,21]. Through computational methods and through the determination of FRAP (ferric reducing antioxidant power) and DPPH (1,1-diphenyl-2-picrylhydrazyl) assays, it was determined that isoespintanol has an important antioxidant capacity [22]. This capacity was also demonstrated in silico assays (discoloration of β-carotene and lipid peroxidation induced by Fe/ascorbate and in the DPPH kinetic assay) [19]. In a mice model of edema induced by carrageenan, isoespintanol showed an inhibitory effect on the production of interleukin 1β [18]. On the other hand, the monoterpene evinced antispasmodic actions on biliary, urinary, and uterine spasms, even with a higher potency than other drugs used in therapy, probably mediated by interference of Ca^2+^ influx to smooth muscle cells [20,23], demonstrated in a rat model of isolated aortic rings that isoespintanol exerts a vasodilator effect, mediated through an ON-dependent signalling pathway. The authors also suggest that the inhibition of Ca^2+^ entry into myocytes could be involved in this mechanism. Finally, in a rat model of ischemia–reperfusion, González-Arbeláez et al. [21], showed that isoespintanol reduces cell mortality, decreases post-ischemia dysfunction, and improves mitochondrial status, probably activating the PKCε-Akt-eNOS signalling pathway.

These previously described actions positioned isoespintanol as a good candidate for treating prediabetes triggered by an unhealthy diet, where oxidative stress and inflammation play a pivotal role.

Therefore, the aim of the present work was to evaluate the possible protective effects of isoespintanol on endocrine–metabolic changes, oxidative stress, and inflammation, induced by administration of a fructose-rich diet in the liver of prediabetic rats.

## 2. Results

### 2.1. Body Weight, Food Intake, and Drink Intake

After 21 days of treatment, rats consuming fructose 10% (F and FI) drank a significantly higher volume compared to C rats (79.87 ± 10.46 and 62.37 ± 7.12 vs. 29.26 ± 2.20 mL/rat/day; *p* < 0.05). However, control rats consumed a significantly higher volume of solid food than F and FI rats (21.81 ± 0.8 vs. 15.86 ± 2.1 and 14.94 ± 1.04 g/rat/day; *p* < 0.05). Despite these differences, their caloric intake was comparable without significant differences (C: 63 ± 5; F: 78 ± 6; FI: 68 ± 4 kcal/rat/day).

### 2.2. Serum Parameters

Although there were no significant differences in glycemia between groups (Table 1), a significant increase in triglyceridemia and the IRX was observed in F group compared to C animals (*p* < 0.05). These changes were significantly reversed with isoespintanol (FI) treatment (Table 1). These data demonstrated that F animals developed dyslipidemia and insulin resistance and that administration of the monoterpene isoespintanol reversed the development of these metabolic–endocrine changes.

### 2.3. Intraperitoneal Glucose Tolerance Test (IGTT)

**Figure 1:**
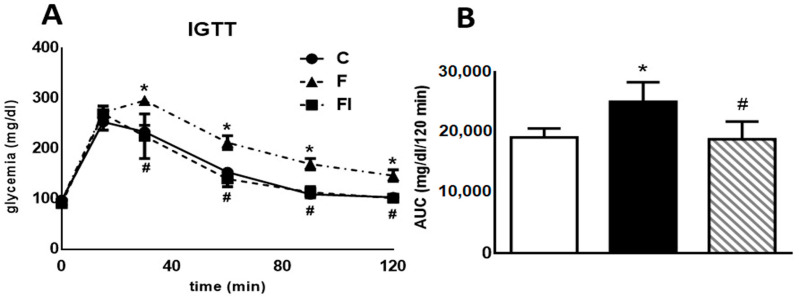
Intraperitoneal glucose tolerance test. (**A**) Blood glucose concentration 0, 30, 60, 90, and 120 min following an IGTT (1.5 g/kg) in C, F, and FI rats. (**B**) AUC expressed in mg/dL/120 min in C (white bars), F (black bars), and FI (lined bars) animals. In both panels, values are means ± SEM. * *p* < 0.05 vs. C; # *p* < 0.05 vs. F; *n* = 6 rats/group.

Figure 1 (both panels) displays data obtained from a high-glucose challenge to rats. Plasma glucose levels measured 30, 60, 90, and 120 min after glucose load were significantly higher in F compared with C animals (*p* < 0.05, Figure 1A). Consequently, the area under the glucose curve (AUC) during the IGTT was also significantly higher in F than in C rats (*p* < 0.05, Figure 1B). Administration of isoespintanol to F rats reversed the increase in both the AUC and the glucose levels at 30, 60, 90, and 120 min (*p* < 0.05).

### 2.4. Liver Parameters

#### 2.4.1. Liver Glycogen Content, p-GSK3β Protein Levels, and Lipogenic Gene Expression

**Figure 2:**
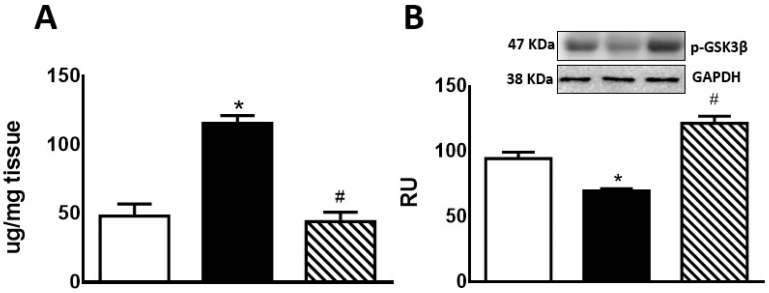
Liver glycogen (**A**) and p-GSK3β protein expression (**B**) by C (white bars), F (black bars), and FI (lined bars) rats. (**B**) Protein levels measured by Western blot in liver homogenates from the different experimental groups. A representative blot of 3 different experiments is shown. GAPDH was used as internal standard. Bars represent means ± SEM expressed in relative units (RU) as the ratio between the protein of interest and GAPDH band intensity. * *p* < 0.0001 vs. C; # *p* < 0.0001 vs. F; *n* = 6 rats/group.

Hepatic glycogen storage was significantly increased in F compared to C rats. In FI rats, there was a significant decrease compared to F group, reaching lower levels than those measured in C rats (Figure 2A). F animals also showed a significant decrease in p-GSK3β protein levels compared to C rats. This effect was reversed by treatment of these rats with isoespintanol (Figure 2B).

**Figure 3:**
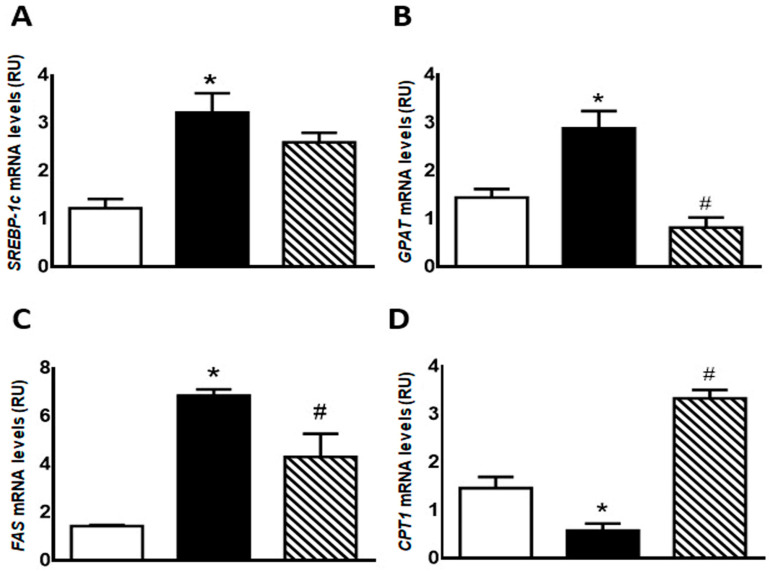
Gene expression of *SREBP-1c* (**A**), *GPAT* (**B**), *FAS* (**C**), and *CPT1* (**D**) was measured by RT-qPCR on liver of C (white bars), F (black bars), and FI (lined bars) rats. Results are expressed as means ± SEM, in relative units (RU). Panel (**A**) * *p* < 0.005 vs. C; panel (**B**) * *p* < 0.02 vs. C, # *p* < 0.002 vs. F; panel (**C**) * *p* < 0.0001 vs. C, # *p* < 0.05 vs. F; panel (**D**) * *p* < 0.005 vs. C, # *p* < 0.001 vs. F; *n* = 6 rats/group.

Regarding lipogenic genes, F rats evinced a significant increase (*p* < 0.05) in *SREBP-1c*, *GPAT*, and *FAS* mRNA levels, while an also significant (*p* < 0.05) reduction was recorded in *CPT1* gene expression. These changes were almost completely reversed in FI rats (Figure 3).

#### 2.4.2. Oxidative Stress (GSH and 3′-Nitrotyrosine) and Inflammation Markers (*TNF-α*, *PAI-1*, and *iNOS* Gene Expression; COX-2 and iNOS Protein Expression)

**Figure 4:**
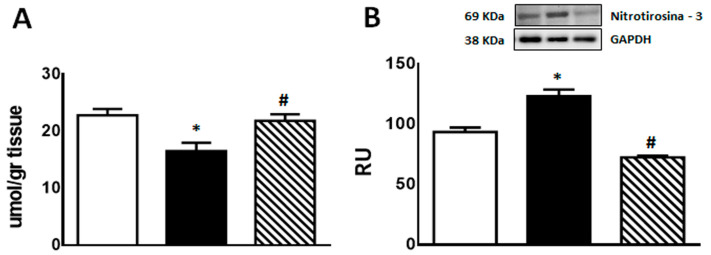
GSH content (**A**) and 3′-nitrotyrosine protein expression (**B**) in liver of C (white bars), F (black bars), and FI (lined bars) rats. A representative blot of 3 different experiments is shown. GAPDH was used as internal standard. Bars represent means ± SEM expressed in relative units (RU) as the ratio between the protein of interest and GAPDH band intensity. Panel (**A**) * *p* < 0.01 vs. C, # *p* < 0.05 vs. F; panel (**B**) * *p* < 0.02 vs. C, # *p* < 0.0001 vs. F; *n* = 6 rats/group.

GSH content was significantly lower in F compared with C animals (*p* < 0.05); however, administration of isoespintanol to F rats increased GSH content to values comparable to those measured in C rats (Figure 4A).

On the other hand, immunoreactive 3′-nitrotyrosine bands (a peroxynitrite production and protein nitration marker) were observed at 69 kDa. 3′-nitrotyrosine protein levels were significantly higher in F than in C group. However, FI rats showed a significant decrease in this marker compared to F animals (Figure 4B).

**Figure 5:**
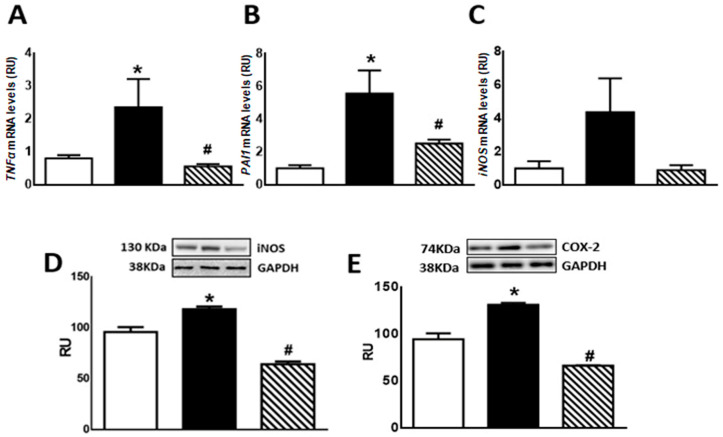
*TNF-α* (**A**), *PAI-1* (**B**), *iNOS* (**C**) gene expression, and iNOS (**D**) and COX-2 (**E**) protein levels in liver of C (white bars), F (black bars), and FI (lined bars) rats. A representative blot of 3 different experiments is shown for iNOS and COX-2. GAPDH was used as internal standard. Bars represent means ± SEM expressed in relative units (RU) as the ratio between iNOS or COX-2 and GAPDH band intensity. Panel (**A**) * *p* < 0.05 vs. C, # *p* < 0.05 vs. F; panel (**B**) * *p* < 0.002 vs. C, # *p* < 0.01 vs. F; panel (**D**) * *p* < 0.002 vs. C, # *p* < 0.0001 vs. F; panel (**E**) * *p* < 0.0001 vs. C, # *p* < 0.0001 vs. F; *n* = 6.

Regarding inflammation markers, F animals showed enhanced *TNF-α* and *PAI-1* mRNA levels as well as COX-2 and iNOS protein levels compared to C rats (*p* < 0.05). Isoespintanol administration to F animals (FI) restored the values to those measured in C group (*p* < 0.05 vs. F) (Figure 5A,B,D,E). A non-significant increase in *iNOS* mRNA levels was recorded in F rats, whereas FI animals evinced a gene expression comparable to C rats (Figure 5C).

#### 2.4.3. Protein Levels of p-Akt and p-eNOS

**Figure 6:**
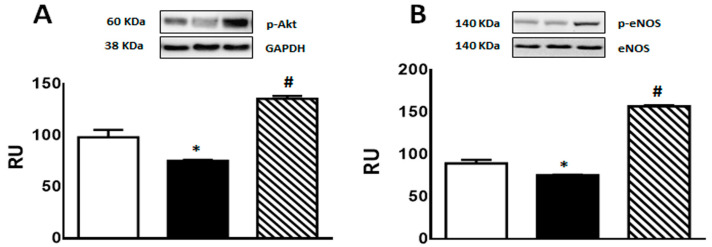
p-Akt (**A**), and p-eNOS (**B**) protein expression determined by Western blot in liver homogenates from C (white bars), F (black bars), and FI (lined bars) rats. A representative blot of 3 different experiments is shown in each case. Bars represent means ± SEM expressed in relative units (RU) as the ratio between the protein of interest and GAPDH or total eNOS band intensity. Panel (**A**) * *p* < 0.01 vs. C; # *p* < 0.0001 vs. F; panel (**B**) * *p* < 0.002 vs. C, # *p* < 0.0001 vs. F; *n* = 6 rats/group.

A significant decrease in p-Akt and p-eNOS protein levels was observed in F rats compared to C group. This effect was reversed by isoespintanol treatment, inducing a significant increase in the expression of both proteins (Figure 6A,B). 

## 3. Discussion

Current results demonstrate for the first time that isoespintanol—a monoterpene isolated from Oxandra cf. xylopioides—administered to rats fed a fructose-rich diet, exerts protective effects, and prevents endocrine–metabolic oxidative stress and inflammatory disturbance triggered by an unhealthy diet. Isoespintanol decreased triglycerides, lipogenic genes, IRX, and enhanced HDL, restored glucose tolerance together with a normalization of liver glycogen, and inflammatory and oxidative stress markers. This study also suggests that Akt/eNOS and Akt//GSK3β signaling pathways play a pivotal role in isoespintanol-mediated protection.

In previous studies employing the rat prediabetes model induced by administration of a fructose-rich diet, we demonstrated enhanced liver oxidative stress and inflammation, together with endocrine–metabolic dysfunction including insulin resistance [8], constituting a triad that self-perpetuates in a vicious pathogenic circle which can be effectively disrupted by antioxidant co-administration [9]. In this sense, isoespintanol has been shown to intervene pharmacologically through different pathways, including oxidative stress and inflammation regulation. Current results demonstrated a clear hepatic antioxidant effect of this monoterpene, thereby increasing GSH level and decreasing nitrotyrosylated protein levels in fructose-fed rats. Rojano et al. [18] demonstrated that isoespintanol exerts a clear anti-inflammatory effect by reducing IL-1β protein production and *IL-1 β* mRNA synthesis in RAW 264.7 macrophages. Our current results suggest that isoespintanol is also able to modulate hepatic inflammation since its administration restored basal expression of *TNF-α*, *PAI1*, and *iNOS* gene expression together with normalized COX-2 and iNOS protein levels. It has been demonstrated that TNF-α interferes with the insulin receptor signaling pathway and with metabolism of glucose transporters, and, consequently, plays a role in the pathophysiology of insulin resistance [24]. Moreover, TNF-α positively regulates PAI-1 expression which, in turn, induces insulin resistance and metabolic abnormalities in liver during proinflammatory processes [25], thus suggesting a common link between TNF-α, insulin resistance, and elevated PAI-1 in obesity [26]. Taken together, our results provide evidence of the importance of regulating inflammation to reverse insulin resistance by using plant products.

Regarding GSK3, it exists in two isoforms, termed α and β. GSK3 is one of the few kinases that is active in its basal state and is inhibited upon phosphorylation at Serine9 in GSK3β and at Serine21 in GSK3α, a phosphorylation mediated by several kinases. In our study, we determined p-GSK3β (Serine9) protein levels. GSK3β is a signaling kinase, involved in regulation of several cellular activities, from metabolism to immune activation [27,28,29,30,31,32]. GSK3β is inhibited by Serine9 phosphorylation, and GSK3β inhibition by this phosphorylation promotes anti-inflammatory gene programs in macrophages [27]. More recently, it was demonstrated that GSK3β phosphorylation at Serine9 leads to an anti-inflammatory response [33]. Since Akt phosphorylates GSK3β on its regulatory serine residue (Serine9), inhibiting its activity [34], increased p-GSK3β (Serine9) in FI animals compared to F rats could be a consequence of the recovery in p-Akt level induced by the phytochemical. These facts could explain the decrease in liver inflammatory markers registered in isoespintanol-treated rats.

Several dysfunctions are characterized by a reduced NO production, associated with reduced phosphorylation of eNOS [35]. Among others, a defect in eNOS phosphorylation has been considered to account for endothelial dysfunction further leading to hypertension and hyperlipidemia [36]. Interestingly, Atochin et al. have shown modulation of Serine1179 phosphorylation as an approach for treating cardiovascular diseases related to diabetes, obesity, metabolic syndrome, hyperlipidemia, and hypertension [37]. eNOS phosphorylation was also shown to be stimulated by phytochemicals, such as polyphenols, through activation of kinases, such as Akt [38,39]. In this sense, isoespintanol was shown to be a good antispasmodic in isolated rat intestine, bladder, and uterus, interfering non-competitively with Ca^2+^ influx into smooth muscle [20]. Further, in rat isolated thoracic aortic rings, isoespintanol showed a vasodilatory effect through NO-dependent pathways [23]. In addition, González Arbeláez et al. have shown that isoespintanol attenuates myocardial dysfunction caused by ischemia and reperfusion in isolated rat hearts and that this cardioprotection involved activation of Akt/eNOS and PKCε signaling pathways [21]. Current results are in line with those previously found and suggest that isoespintanol also modulates the Akt/eNOS pathway in the liver of prediabetic rats.

Fructose-fed rats showed significantly lower levels of p-Akt (phosphorylated at Serine473) together with an increase in hepatic markers of lipogenesis [8]. It is known that Akt can be activated by phosphorylation at Serine473 by mTORC2 [40]. Also, hepatic fat accumulation in rats with dietary-induced non-alcoholic fatty liver disease was shown to be accompanied by reduced phosphorylation of Akt [41,42]. Additionally, pharmacological inhibition of Akt led to fat accumulation in rat liver [41]. By contrast, Akt activation could alleviate liver steatosis in several models of non-alcoholic fatty liver disease and diabetic mice [43,44,45,46]. Current results clearly demonstrated that isoespintanol prevents the changes recorded in p-Akt and, in turn, this prevention could play a key role in the regulation of lipid homeostasis in the liver of fructose-fed rats.

**Figure 7:**
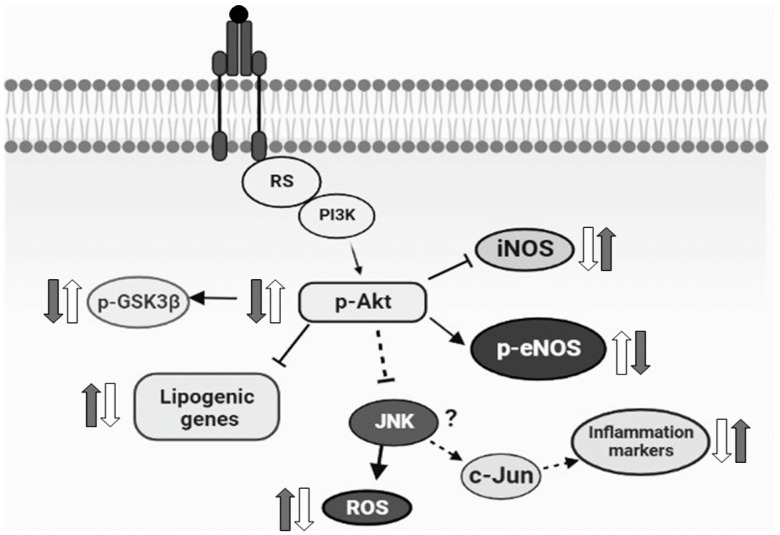
Schematic diagram representing the possible mechanism of the action of isoespintanol. Based on the current results, we proposed that the increased lipogenic gene expression, inflammatory markers, and oxidative stress measured in HFD animals (grey arrows) were reversed by isoespintanol (white arrows), probably modulating Akt/eNOS and Akt/GSK3β signaling pathways.

In conclusion, our current results demonstrated for the first time that the monoterpene isoespintanol prevented endocrine–metabolic, inflammatory, and oxidative stress disturbances triggered by an unhealthy diet (rich in fructose), probably modulating Akt/eNOS and Akt/GSK3β signaling pathways, at least in our rat model (Figure 7). Even when extrapolation from a surrogate model to human clinical research is useful, it must be justified by means of a variety of theoretical and experimental considerations. In this sense, current results positioning isoespintanol as a possible new therapeutic tool to treat diabetes at an early stage of development (prediabetes) merit further research to validate these findings in human subjects.

## 4. Materials and Methods

### 4.1. Chemicals and Drugs

All reagents of the purest available grade were provided by Sigma Chemical Co. (St. Louis, MO, USA).

**Figure 8:**
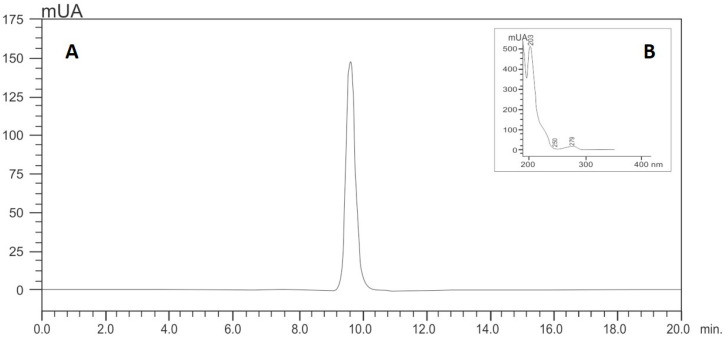
HPLC (**A**) and UV profile (**B**) of isoespintanol.

Isoespintanol was obtained from Oxandra cf. xylopioides, as previously detailed [18]. Briefly, dry and ground leaves of this plant product were extracted with petroleum ether by percolation and dried by rotary evaporation. The extract was subjected to various chromatographic columns by gravity, eluting with hexane–dichloromethane mixtures and, finally, recrystallizing. The structure of the compound was established based on interpretation of its NMR and MS data [18]. The purity of this sample was assessed by HPLC analyses, for which isoespintanol was dissolved in 10.0 mL of ethanol (at a final concentration of 500 mg/L) under ultrasound for 15 min. Its purity was quantified using HPLC-DAD (Shimadzu Prominence^®^, Tokyo, Japan) according to the modified method proposed by Ultee et al. [47]: isocratic elution with methanol/water (60:40); flow rate of 1.0 mL/min; with a LiChrospher^®^ 100 RP-18 column (5 µm, 250 × 4 mm) (Merck, Darmstadt, Germany) as stationary phase thermostatted at 35 °C and injection volume of 20 µL. The retention time of isoespintanol was 9.28 min and its purity >99% when monitoring at 277 nm (Figure 8). For the present evaluation, purified crystalline solid isoespintanol was dissolved in dimethylsulphoxide (DMSO) at a concentration of 21 mg/mL as mother solutions.

### 4.2. Experimental Groups

**Figure 9:**
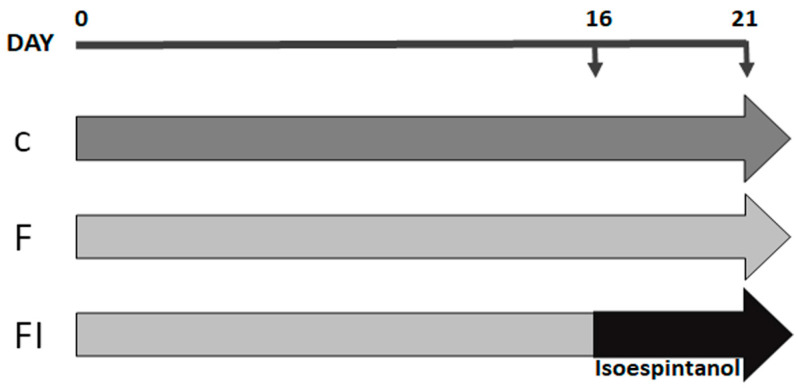
Flowchart illustrating the study design. Animals were maintained for 21 days with standard commercial diet (C group, dark grey arrow), standard commercial diet with the addition of 10% fructose (*w*/*v*) in the drinking water (F group, light grey arrow), and F plus a daily intraperitoneal injection of isoespintanol (10 mg/kg of body weight/day in DMSO, final volume 100 µL), during the last 5 days of treatment (black arrow) (FI group).

Adult male Sprague Dawley rats (250–300 g) were maintained in treatment for 21 days divided into 3 experimental groups: C: standard commercial diet; F: standard commercial diet with the addition of 10% fructose (*w*/*v*) in the drinking water; and FI: treatment equal to the above plus a daily intraperitoneal injection of isoespintanol (10 mg/kg of body weight/day in DMSO, final volume 100 µL), during the last 5 days of treatment (Figure 9).

Dose employed, application route, and treatment time were chosen after testing different conditions. The safety profile of isoespintanol and liver function were determined previously by measuring alanine transaminase (ALT), aspartate transaminase (AST), alkaline phosphatase (ALP), and gamma-glutamyl transferase (GGT). Likewise, in previous trials, the innocuousness of DMSO as a vehicle in control animals was verified. Animals were kept in an environment with constant temperature and humidity (23 ± 1 °C, 50% humidity) and controlled 12 h light/dark cycles. During treatment, water and food consumed were recorded every second day and body weight weekly. After 21 days, animals were killed by decapitation. Blood and liver samples were obtained to conduct the corresponding determinations. Experiments were performed according to “Ethical principles and guidelines for experimental animals” (3rd Edition, 2005) by the Swiss Academy of Medical Sciences (http://www.aaalac.org). All the protocols were approved by the Animal Welfare Committee (CICUAL) of the La Plata School of Medicine, UNLP (T06-01-2022).

### 4.3. Determination of Serum Parameters

Non-fasting blood glucose was measured using test strips (Accu-Chek Performa Nano System, Roche Diagnostics, Mannheim, Germany); triglyceridemia by an enzymatic colorimetric method (TG Color GPO/PAP AA kit; Wiener Lab, Rosario, Argentina); total cholesterol and HDL-cholesterol levels by an enzymatic method (Colestat Enz AA kit, Wiener Lab, Argentina), and non-HDL cholesterol levels were calculated by the difference between total and HDL-cholesterol. Insulin resistance was determined through the IR index (TG/HDL-cholesterol) [48].

### 4.4. Intraperitoneal Glucose Tolerance Test (IGTT)

In the morning (07:00 h) of the experimental day, rats were weighed after 12 h fasting. Subsequently, they were injected i.p. with 200 µL of a freshly prepared glucose solution in sterile saline buffer (1.5 g/kg of body weight) [49]. Blood samples were obtained from the retro-orbital plexus before (baseline; t = 0 min) and 15, 30, 60, 90, and 120 min after glucose challenge. In these samples, glucose concentration was measured with test strips (Accu-Chek Performa Nano System, Roche Diagnostics, Mannheim, Germany). The data were then used to further calculate the area under the curve (AUC) of circulating glucose values expressing it in mg/dL/120 min.

### 4.5. Determination of Liver Parameters

#### 4.5.1. Glycogen Estimation

Liver samples (0.5 g) were placed in 1 mL KOH 33% and incubated at 100 °C for 20 min. After that, samples were cooled to 0 °C and incubated with 1.25 mL of ethanol 96% at 4 °C for 72 h. Then, samples were centrifuged at 700× *g* for 20 min and the precipitate was resuspended in 1 mL of distilled water plus 3 mL of anthrone reagent 100 mg/% (in H_2_SO_4_ 84%) and incubated at 100 °C for 20 min. Absorbance was measured at 620 nm and each reading was interpolated onto a reference curve of known glycogen concentrations. The results were expressed as µg glycogen/mg tissue.

#### 4.5.2. Estimation of Reduced Glutathione (GSH) Content

Hepatic GSH content was determined by the Ellman method [50]. Liver pieces were homogenized in phosphate-K buffer (K2HPO4 10 mM, KCl 11.5 g/L, and pH 7.4, buffer/tissue ratio 1:4). Then, an aliquot of the homogenate was centrifuged at 800× *g* for 20 min. Then, 90 µL of TCA 28% was added to 410 µL of the supernatant, and incubated on ice for 10 min, followed by centrifugation at 4500× *g* for 15 min. Finally, the reaction mixture was carried out in duplicate: 450 µL of TCA 5%, 1 mL of Tris-HCl buffer (pH 8.9) 0.01 M, and 25 µL of dithionitrobenzoic acid (0.4% in methanol) 10 mM were added to 50 µL of the resulting supernatant. The absorbance was measured by a photometric method at 412 nm against a TCA and Tris blank. The results were expressed as µmol GSH/g tissue.

#### 4.5.3. Protein Expression by Western Blot

Liver samples from C, F, and FI rats were used to measure protein levels of p-eNOS (Serine1179), iNOS, p-Akt (Serine473), p-GSK3β (Serine9), COX-2, and protein nitrosylation (3′-nitrotyrosine). The protein concentration of the samples was measured using the Biorad protein assay reagent. The tissue was homogenized in ice-cold RIPA buffer (sucrose 300 mM, DTT 1 mM, EGTA 4 mM, TRIS 20 mM pH 7.4, 1% Triton X100, 10% protease cocktail, NaF 25 μM, and Orthovanadate 1 μmol/L). The buffer was added to each sample in a 1:3 ratio (*w*/*v*), heated at 95 °C for 10 min, and centrifuged at 12,000× *g* for 15 min at 4 °C. The supernatant proteins were resolved on SDS-PAGE and transferred to a PVDF membrane at constant 10 V for 30 min using transfer buffer (Tris base 48 mM, glycine 39 mM, SDS 1.3 mM, methanol 20%, and pH 9.2). Equal loading of samples was confirmed by Ponceau S staining. The membranes were blocked with 5% fatty-acid-free milk in TBS buffer (Tris 20 mM, NaCl 500 mM, and pH 7.5) with Tween 20 0.1%. After that, the membranes were washed and incubated with the corresponding primary antibody diluted 1:1000 in TBS-T and BSA 1% overnight at 4 °C. GAPDH was used as the internal standard. The membranes were washed 4 times for 10 min in TBS-T prior to the addition of HRP-conjugated anti-rabbit secondary antibody (1:5000) and the protein bands were analyzed by a chemiluminescent system (ECL Plus; Amersham Biosciences, Amersham, UK). The total protein signal was used as a loading control. The membranes were dried and scanned, and the bands quantified using Image Studio Digits 3.1 software.

#### 4.5.4. Total RNA Isolation and Analysis of Gene Expression by Real-Time PCR (qPCR)

For total hepatic RNA isolation, TRIzol Reagent (Gibco-BRL, Rockville, MD, USA) was used. Integrity and purity were evaluated by 1% agarose-formaldehyde gel electrophoresis and by measuring 260/280 nm absorbance ratio. To avoid DNA contamination, DNase I (Gibco-BRL) digestion was used. Reverse transcription-PCR was performed using SuperScript III (Gibco-BRL) and 50 ng total RNA as a template.

Real-time PCR was performed using a Mini Opticon Real-Time PCR Detector Separate MJR (BioRad, Hercules, CA, USA) (SYBR Green I was employed as a fluorescent dye). Ten ng of cDNA was amplified in a reaction mixture containing 0.6 µM of each primer, MgCl_2_ 3 mM, dNTPs 0.3 mM, and 0.2 µL Platinum Taq DNA polymerase 6 U/µL (Invitrogen, Buenos Aires, Argentina). Optimal parameters were empirically defined. Oligonucleotide primers are listed on Table 2.

All amplicons were designed in a size range of 90 to 250 bp and β-actin was used as a housekeeping gene. Purity and specificity of products were verified by melting curves. Product length and PCR specificity were further checked by 2% (*w*/*v*) agarose gel electrophoresis and ethidium bromide staining. Data are expressed as relative gene expression (relative units, RU) after normalization to the β-actin housekeeping gene using Qgene96 and LineRegPCR 11.0 software, as described elsewhere [51].

### 4.6. Statistical Data Analysis

Statistical analysis was performed by ANOVA followed by Dunnett’s test for multiple comparisons (GraphPad Prism 6.01). Bartlett’s test was used to assess the variance homogeneity. Results are expressed as mean ± SEM for the indicated number of observations. Differences were considered significant when *p* < 0.05.

## Figures and Tables

**Table 1:** Serum parameters.

Plasma Parameters	C	F	FI	*p* Value
Glucose (mg/dL)	134.8 ± 5.8	133.6 ± 6.7	133.1 ± 4.4	NS
Triglyceride (mg/dL)	96.3 ± 16.1	264.2 ± 21.2 *	79.9 ± 9 #	*# *p <* 0.0001
Chol-HDL (mg/dL)	50.3 ± 5.7	43.4 ± 4.8	52.1 ± 2.8	NS
IRX (TG/HDL)	2.0 ± 0.3	6.4 ± 0.8 *	1.6 ± 0.3 #	*# *p <* 0.001
Cholesterol-Total (mg/dL)	64.0 ± 5.4	78.0 ± 6.7	86.8 ± 6.4	NS
Cholesterol-NoHDL (mg/dL)	13.7 ± 2.0	34.6 ± 2.7 *	34.7 ± 6.0	* *p <* 0.0001

Values are expressed as mean ± SEM. * *p* vs. C; # *p* vs. F; *n* = 6 rats/group.

**Table 2:** Rat-specific primers used for real-time PCR analyses. FW: forward primer and RV: reverse primer.

Gene	GeneBank^®^	Sequences
*SREBP-1c*	XM_213329.6	FW 5′-TTTCTTCGTGGATGGGGACT-3′ RV 5′-CTGTAGATATCCAAGAGCATC-3′
*FAS*	NM_017332.1	FW 5′-GTCTGCAGCTACCCACCCGTG-3′ RV 5′-CTTCTCCAGGGTGGGGACCAG-3′
*GPAT-1*	NM_017274.1	FW 5′-GACGAAGCCTTCCGAAGGA-3′ RV 5′-GACGAAGCCTTCCGAAGGA-3′
*PAI-1*	NM_012620.1	FW 5′-CCACGGTGAAGCAGGTGGACT-3′ RV 5′-TGCTGGCCTCTAAGAAGGGG-3′
*TNF-α*	NM_012675.3	FW 5′-GGCATGGATCTCAAAGACAACC-3′ RV 5′-CAAATCGGCTGACGGTGTG-3′
*CPT1*	NM_031559.2	FW: 5′ GGGCGGTACTTCAAGGTCTGG 3′ RV: 5′ GTCTGCCGACACTTTGCCCA 3′
*eNOS*	NM_021838.2	FW: 5′ GCTGGGGGATCAGCAACGCT 3′ RV: 5′ GCGGGTCAAAGGACCAGGGC 3′
*iNOS*	NM_012611.3	FW: 5′ GAAGCTCAGCCGCACCACCC 3′ RV: 5′ CAGGGCCGTCTGGTTGCCTG 3′
*β-ACTIN*	NM_031144.2	FW 5′-AGAGGGAAATCGTGCGTGAC-3′ RV 5′-CGATAGTGATGACCTGACCGT-3′

## Data Availability

Data is contained within the article.

## Abbreviations

Alanine transaminase (ALT), alkaline phosphatase (ALP), area under the glucose curve (AUC), carnitine palmitoyl transferase 1 (CPT1), Cyclooxygenase-2 (COX-2), 1,1-diphenyl-2-picrylhydrazyl (DPPH), endothelial nitric oxide synthase (eNOS), fatty acid synthase (FAS), ferric reducing antioxidant power (FRAP), gamma-glutamyl transferase (GGT), glyceraldehyde-3-phosphate dehydrogenase (GAPDH), glycerol-3-phosphate acyltransferase (GPAT), glycogen synthase kinase-3β (GSK3β), high-fructose corn syrup (HFCS), high-fructose diet (HFD), homeostasis model assessment (HOMA), inducible nitric oxide synthase (iNOS), insulin resistance index (IRX), intraperitoneal glucose tolerance test (IGTT), plasminogen activator-1 (PAI-1), protein kinase B (Akt), reduced glutathione (GSH), sterol regulatory element binding protein (SREBP-1c), tumor necrosis factor α (TNF-α), and type 2 diabetes (T2D).

## References

[1] Lovic D., Piperidou A., Zografou I., Grassos H., Pittaras A., Manolis A. (2020). The Growing Epidemic of Diabetes Mellitus. Curr. Vasc. Pharmacol..

[2] American Diabetes Association (2019). Introduction: Standards of Medical Care in Diabetes—2019. Diabetes Care.

[3] Carvallo P., Carvallo E., Barbosa-Da-Silva S., Mandarim-De-Lacerda C., Hernández A., Del Sol M. (2019). Metabolic Effects of Excessive Fructose Consumption Added. Int. J. Morphol..

[4] Nier A., Brandt A., Conzelmann I.B., Özel Y., Bergheim I. (2018). Non-alcoholic fatty liver disease in overweight children: Role of fructose intake and dietary pattern. Nutrients.

[5] Czerwonogrodzka-Senczyna A., Rumińska M., Majcher A., Credo D., Jeznach-Steinhagen A., Pyrżak B., Pokorski M. (2019). Fructose Consump-tion and Lipid Metabolism in Obese Children and Adolescents. Advances in Experimental Medicine and Biology.

[6] Olson E., Suh J.H., Schwarz J.M., Noworolski S.M., Jones G.M., Barber J.R., Erkin-Cakmak A., Mulligan K., Lustig R.H., Mietus-Snyder M. (2022). Effects of Isocaloric Fructose Restriction on Ceramide Levels in Children with Obesity and Cardi-ometabolic Risk: Relation to Hepatic De Novo Lipogenesis and Insulin. Sensitivity. Nutrients.

[7] Bence K.K., Birnbaum M.J. (2021). Metabolic drivers of non-alcoholic fatty liver disease. Mol. Metab..

[8] Castro M.C., Villagarcía H.G., Román C.L., Maiztegui B., Flores L.E., Schinella G.R., Massa M.L., Francini F. (2022). Chronolog-ical appearance of endocrine and metabolic dysfunctions induced by an unhealthy diet in rats. Medicina.

[9] Castro M.C., Massa M.L., Arbeláez L.G., Schinella G., Gagliardino J.J., Francini F. (2015). Fructose-induced inflammation, insulin resistance and oxidative stress: A liver pathological triad effectively disrupted by lipoic acid. Life Sci..

[10] Francini F., Castro M.C., Gagliardino J.J., Massa M.L. (2009). Regulation of liver glucokinase activity in rats with fructose-induced insulin resistance and impaired glucose and lipid metabolism. Can. J. Physiol. Pharmacol..

[11] Francini F., Castro M.C., Schinella G., García M.E., Maiztegui B., Raschia M.A., Gagliardino J.J., Massa M.L. (2010). Changes in-duced by a fructose-rich diet on hepatic metabolism and the antioxidant system. Life Sci..

[12] Maiztegui B., Borelli M.I., Raschia M.A., Del Zotto H., Gagliardino J.J. (2009). Islet adaptive changes to fructose-induced insulin resistance: Beta-cell mass, glucokinase, glucose metabolism, and insulin secretion. J. Endocrinol..

[13] Newman D.J., Cragg G.M. (2020). Natural Products as Sources of New Drugs over the Nearly Four Decades. J. Nat. Prod..

[14] Mahdavi A., Bagherniya M., Mirenayat M.S., Atkin S.L., Sahebkar A. (2021). Medicinal Plants and Phytochemicals Regulating Insulin Resistance and Glucose Homeostasis in Type 2 Diabetic Patients: A Clinical Review. Adv. Exp. Med. Biol..

[15] Bessell E., Fuller N.R., Markovic T.P., Burk J., Picone T., Hendy C., CTan M.M., Caterson I.D. (2019). Effects of alpha-cyclodextrin on cholesterol control and Compound K on glycaemic control in people with pre-diabetes: Protocol for a Phase III randomized controlled trial. Clin. Obes..

[16] Naseri K., Saadati S., Sadeghi A., Asbaghi O., Ghaemi F., Zafarani F., Li H.-B., Gan R.-Y. (2022). The efficacy of Ginseng (Panax) on Human Prediabetes and Type 2 Diabetes Mellitus: A Systematic Review and Meta-Analysis. Nutrients.

[17] Bessell E., Fuller N.R., Markovic T.P., Lau N.S., Burk J., Hendy C., Picone T., Li A., Caterson I.D. (2020). Effects of α-cyclodextrin on cholesterol control and hydrolyzed ginseng extract on glycemic control in people with prediabetes: A randomized clinical trial. JAMA Netw. Open.

[18] Rojano B., Pérez E., Figadère B., Martin M.T., Recio M.C., Giner R., Ríos J.L., Schinella G., Sáez J. (2007). Constituents of Oxandra cf. xylopioides with anti-inflammatory activity. J. Nat. Prod..

[19] Rojano B., Gaviria C., Gil M., Saez J., Guillermo S., Tournier H. (2008). Antioxidant Activity of the Isoespintanol in Different Media. Vitae.

[20] Gavilánez Buñay T.C., Colareda G.A., Ragone M.I., Bonilla M., Rojano B.A., Schinella G.R., Consolini A.E. (2018). Intestinal, urinary and uterine antispasmodic effects of isoespintanol, metabolite from Oxandra xylopioides leaves. Phytomedicine.

[21] González Arbeláez L.F., Ciocci Pardo A., Fantinelli J.C., Rojano B., Schinella G.R., Mosca S.M. (2020). Isoespintanol, a mono-terpene isolated from oxandra cf xylopioides, ameliorates the myocardial ischemia-reperfusion injury by AKT/PKCε/eNOS-dependent pathways. Naunyn-Schmiedeberg Arch. Pharmacol..

[22] Rojano B., Saez J., Schinella G., Quijano J., Vélez E., Gil A., Notario R. (2008). Experimental and theoretical determination of the antioxidant properties of isoespintanol (2-isopropyl-3,6-dimethoxy-5-methylphenol). J. Mol. Struct..

[23] Rinaldi G.J., Rojano B., Schinella G., Mosca S.M. (2019). Participation of NO in the vasodilatory action of Isoespintanol. Vitae.

[24] Borst S.E. (2004). The role of TNF-α in insulin resistance. Endocrine.

[25] Kaji H. (2016). Adipose Tissue-Derived Plasminogen Activator Inhibitor-1 Function and Regulation. Compr. Physiol..

[26] Samad F., Uysal K.T., Wiesbrock S.M., Pandey M., Hotamisligil G.S., Loskutoff D.J. (1999). Tumor necrosis factor α is a key component in the obesity-linked elevation of plasminogen activator inhibitor 1. Proc. Nat. Acad. Sci. USA.

[27] Beurel E., Michalek S.M., Jope R.S. (2010). Innate and adaptive immune responses regulated by glycogen synthase kinase-3 (GSK3). Trends Immunol..

[28] Cohen P., Goedert M. (2004). GSK3 inhibitors: Development and therapeutic potential. Nat. Rev. Drug Discov..

[29] McManus E.J., Sakamoto K., Armit L.J., Ronaldson L., Shpiro N., Marquez R., Alessi D.R. (2005). Role that phosphorylation of GSK3 plays in insulin and Wnt signalling defined by knockin analysis. EMBO J..

[30] Rayasam G.V., Tulasi V.K., Sodhi R., Davis J.A., Ray A. (2009). Glycogen synthase kinase 3: More than a namesake. Br. J. Pharmacol..

[31] Wang H., Brown J., Martin M. (2011). Glycogen synthase kinase 3: A point of convergence for the host inflammatory response. Cytokine.

[32] Zhou H., Wang H., Ni M., Yue S., Xia Y., Busuttil R.W., Kupiec-Weglinski J.W., Lu L., Wang X., Zhai Y. (2018). Glycogen syn-thase kinase 3β promotes liver innate immune activation by restraining AMP-activated protein kinase activation. J. Hepatol..

[33] Cortés-Vieyra R., Silva-García O., Gómez-García A., Gutiérrez-Castellanos S., Álvarez-Aguilar C., Baizabal-Aguirre V.M. (2021). Glycogen Synthase Kinase 3β Modulates the Inflammatory Response Activated by Bacteria, Viruses, and Parasites. Front. Immunol..

[34] Jope R.S., Yuskaitis C.J., Beurel E. (2007). Glycogen Synthase Kinase-3 (GSK3): Inflammation, Diseases, and Therapeutics. Neurochem. Res..

[35] Maia A.R., Batista T.M., Victorio J.A., Clerici S.P., Delbin M.A., Carneiro E.M., Davel A.P. (2014). Taurine supplementation reduces blood pressure and prevents endothelial dysfunction and oxidative stress in post-weaning protein-restricted rats. PLoS ONE.

[36] Kolluru G.K., Siamwala J.H., Chatterjee S. (2010). eNOS phosphorylation in health and disease. Biochimie.

[37] Atochin D.N., Wang A., Liu VW T., Critchlow J.D., Dantas AP V., Looft-Wilson R., Murata T., Salomone S., Shin H.K., Ayata C. (2007). The phosphorylation state of eNOS modulates vascu-lar reactivity and outcome of cerebral ischemia in vivo. J. Clin. Investig..

[38] Gerardi G., Cavia-Saiz M., Rivero-Pérez M.D., González-SanJosé M.L., Muñiz P. (2019). Modulation of Akt-p38-MAPK/Nrf2/SIRT1 and NF-κB pathways by wine pomace product in hyperglycemic endothelial cell line. J. Funct. Foods..

[39] Gerardi G., Cavia-Saiz M., Rivero-Pérez M.D., González-SanJosé M.L., Muñiz P. (2020). The protective effects of wine pomace products on the vascular endothelial barrier function. Food. Funct..

[40] Zoncu R., Efeyan A., Sabatini D.M. (2011). MTOR: From growth signal integration to cancer, diabetes and ageing. Nat. Rev. Mol. Cell Biol..

[41] Han J.W., Zhan X.R., Li X.Y., Xia B., Wang Y.Y., Zhang J., Li B.X. (2010). Impaired PI3K/Akt signal pathway and hepatocellu-lar injury in high-fat fed rats. World J. Gastroenterol..

[42] Castro M.C., Villagarcía H., Nazar A., Arbeláez L.G., Massa M.L., Del Zotto H., Ríos J.L., Schinella G.R., Francini F. (2020). Cacao extract enriched in polyphenols prevents endocrine-metabolic disturbances in a rat model of prediabetes triggered by a sucrose rich diet. J. Ethnopharmacol..

[43] Bijl N., Sokolović M., Vrins C., Langeveld M., Moerland P.D., Ottenhoff R., Van Roomen C.P.A.A., Claessen N., Boot R.G., Aten J. (2009). Modulation of glycosphingolipid metabolism significantly im-proves hepatic insulin sensitivity and reverses hepatic steatosis in mice. Hepatology.

[44] Zhang Y., Hai J., Cao M., Zhang Y., Pei S., Wang J., Zhang Q. (2013). Silibinin ameliorates steatosis and insulin resistance during non-alcoholic fatty liver disease development partly through targeting IRS-1/PI3K/Akt pathway. Int. Immunopharmacol..

[45] Wang C., Chi Y., Li J., Miao Y., Li S., Su W., Jia S., Chen Z., Du S., Zhang X. (2014). FAM3A activates PI3K p110α/Akt signaling to ameliorate hepatic gluconeogenesis and lipogenesis. Hepatology.

[46] Zeng T., Zhang C.L., Zhao N., Guan M.J., Xiao M., Yang R., Zhao X.L., Yu L.H., Zhu Z.P., Xie K.Q. (2018). Impairment of Akt activity by CYP2E1 mediated oxidative stress is involved in chronic ethanol-induced fatty liver. Redox Biol..

[47] Ultee A., Bennik MH J., Moezelaar R. (2022). The phenolic hydroxyl group of carvacrol is essential for action against the food-borne pathogen Bacillus cereus. Appl. Environ. Microbiol..

[48] Laws A., Reaven G.M. (1992). Evidence for an independent relationship between insulin resistance and fasting plasma HDL-cholesterol, triglyceride and insulin concentrations. J. Intern. Med..

[49] De Vogel-Van Den Bosch J., Hoeks J., Timmers S., Houten S.M., Van Dijk P.J., Boon W., Van Beurden D., Schaart G., Kersten S., Voshol P.J. (2011). The effects of long-or medium-chain fat diets on glucose tolerance and myocellular content of lipid intermediates in rats. Obesity.

[50] Sedlak J., Lindsay R.H. (1968). Estimation of total, protein-bound, and nonprotein sulfhydryl groups in tissue with Ellman’s rea-gent. Anal. Biochem..

[51] Pfaffl M.W. (2001). A new mathematical model for relative quantification in real-time RT-PCR. Nucleic Acids Res..

